# RIPK1 suppresses apoptosis mediated by TNF and caspase-3 in intervertebral discs

**DOI:** 10.1186/s12967-019-1886-3

**Published:** 2019-04-27

**Authors:** Xubin Qiu, Ming Zhuang, Ziwen Lu, Zhiwei Liu, Dong Cheng, Chenlei Zhu, Jinbo Liu

**Affiliations:** 1 Department of Spine, The Third Affiliated Hospital of Soochow University, 185 Juqian Street, Tianning District, Changzhou, 213003 Jiangsu China; 2 School of Pharmacy, Jiangsu University, Zhenjiang, 212013 Jiangsu China

**Keywords:** Intervertebral disc, Degeneration, Apoptosis, RIPK1

## Abstract

**Background:**

Low back pain has become a serious social and economic burden and the leading cause of disability worldwide. Among a variety of pathophysiological triggers, intervertebral disc (IVD) degeneration plays a primary underlying role in causing such pain. Specifically, multiple independent endplate changes have been implicated in the initiation and progression of IVD degeneration.

**Methods:**

In this study, we built a signaling network comprising both well-characterized IVD pathology-associated proteins as well as some potentially correlated proteins that have been associated with one or more of the currently known pathology-associated proteins. We then screened for the potential IVD degeneration-associated proteins using patients’ normal and degenerative endplate specimens. Short hairpin RNAs for receptor interacting serine/threonine kinase 1 (*RIPK1*) were constructed to examine the effects of *RIPK1* knockdown in primary chondrocyte cells and in animal models of caudal vertebra intervertebral disc degeneration in vivo.

**Results:**

RIPK1 was identified as a potential IVD degeneration-associated protein based on IVD pathology-associated signaling networks and the patients’ degenerated endplate specimens. Construction of the short hairpin RNAs was successful, with short-term *RIPK1* knockdown triggering inflammation in the primary chondrocytes, while long-term knockdown triggered apoptosis through cleavage of the caspase 3 pathway, down-regulated NF-κB and mitogen-activating protein kinase (MAPK)s cascades, and decreased cell survival and inflammation. Animal models of caudal vertebra intervertebral disc degeneration further demonstrated that apoptosis was induced by up-regulation of tumor necrosis factor (TNF) accompanied by down-regulation of NF-κB and MAPKs cascades that are dependent on caspase and RIPK1.

**Conclusions:**

These results provide proof-of-concept for developing novel therapies to combat IVD degeneration through interfering with RIPK1-mediated apoptosis signaling pathways especially in patients with RIPK1 abnormality.

**Electronic supplementary material:**

The online version of this article (10.1186/s12967-019-1886-3) contains supplementary material, which is available to authorized users.

## Background

The social and economic burden of low back pain continues to escalate due mainly to changes in lifestyle and demographic profile, with such pain now the leading cause of disability worldwide [1]. Although many different pathophysiological causes might trigger low back pain, it has been demonstrated that intervertebral disc (IVD) degeneration plays a primary underlying role. By connecting the neighboring vertebrae of the spinal column and allowing slight movement flexibility of the spine, IVDs act to absorb axial compressive forces and facilitate load transmission. IVDs comprise an annulus fibrosus surrounding the central nucleus pulposus, the fibrocartilage, and endplates.

Decades of research into IVDs have revealed many secrets of structure, function, and molecular mechanisms; however, how these large structures can survive and function even under the most difficult physiological conditions remains an enigma. The lumbar spine carries considerable forces and has no dedicated blood supply, thus the vertebral endplate plays an important role in balancing the contradictory functions of permeability for nutrients to diffuse between disc cells and capillaries in the adjacent vertebra and vascularized tissues with sufficient strength to prevent damage or fracture. In addition, endplates function to absorb and separate significant pressure from the spine’s mechanical burden and prevent the nucleus pulposus from bulging into the adjacent vertebrae and are essential for disc metabolism.

Some studies have suggested that IVD degeneration is closely correlated with the state of the vertebral endplates [2, 3], which often show significant morphological changes with aging-related IVD degenerations [4]. The biochemical changes in endplates have been reported extensively, from normal to different degenerative conditions [4]. Ariga et al. showed that increased apoptosis in the cartilaginous endplate with age resulted in markedly decreased cell density and destruction of the cartilaginous endplate [5], followed by the structure of the cartilaginous endplate beginning to disappear. Multiple independent factors can cause the initiation and progression of degeneration through endplate changes (reviewed in [6–8]). The process is a chain of biochemical, cellular, structural, and functional changes in the endplates, with mechanical stress, nutrient supply, osmotic and ionic environments, hormones, cytokines, growth factors, and matrix molecules all reported to affect disc cell degeneration, and many other pathological causes still to be explored.

Receptor interacting serine/threonine kinase 1 (RIPK1) is involved in Toll-like receptor (TLR), tumor necrosis factor (TNF), interferon, and interleukin (IL)1α signaling pathways [9–14]. Several studies found that activated RIPK1 can associate with RIPK3 to induce mixed lineage kinase domain like pseudokinase (MLKL)-dependent necroptosis and production of inflammatory cytokines or recruit Fas-associated protein with death domain (FADD) and activate caspase-8 to induce apoptosis following DNA damage or TLR signaling. In addition, it participates in the nuclear factor kappa-light-chain-enhancer of activated B cells (NF-κB) activation independent of its kinase activity. Abnormal activities of RIPK1 have been indicated in several disease processes, such as ischemic injuries, chronic and acute inflammatory diseases, axonal degeneration, neutrophilic dermatosis, autoinflammatory and autoimmune pathology, and cancers [14]. Due to RIPK1′s role in regulating necroptosis and apoptosis, it has gained interests as a treatment target for the RIPK1-dependent diseases already mentioned. In addition, the cumulative effects of various functions performed by RIPK1 may collaboratively contribute to molecular pathologies of autoimmune, degenerative, and inflammatory diseases.

This study sought to identify potential molecular pathogenic markers of IVD degeneration through building signaling networks based on known pathways important in IVD degeneration and to screen against the network using clinical specimens to reveal the underlying mechanisms of such molecules.

## Materials and methods

### Antibodies and other reagents

The following antibodies were used in this study: RIPK1 (Cell Signaling Technology, Danvers, MA), p-IKKα/β^Ser176/Ser180^ (Thermo Fisher Scientific, Waltham, MA), p-JNK^Thr183^ (Abcam, Cambridge, MA), p-IKKα/β^Ser176/180^, p-p38^Thr180/Tyr182^, β-actin, and cleaved caspase 3 (Cell Signaling Technology). Other reagents purchased are as follows: 5-bromo-4-chloro-3-indolyl-β-d-galactopyranoside) (X-Gal), zVAD (*N*-benzyloxycarbonyl-Val-Ala-Asp (O-Me) fluoromethyl ketone), and necrostatin-1 (Nec1) from Sigma-Aldrich (St. Louis, MO), cycloheximide (CHX) from Santa Cruz Biotech (Dallas, Texas), collagenase II from Thermo Fisher Scientific, BCA Protein Assay Kit from Beyotime Biotechnology (Shanghai, China), HiScript Q Select RT SuperMix for qPCR and AceQ qPCR Probe Master Mix from Vazyme Biotech (Nanjing, China), and all remaining reagents from Sigma-Aldrich unless otherwise specified.

### Building a signaling network representing intervertebral disc diseases with pathology-associated proteins

Reported pathology-associated proteins in IVD diseases were used as signaling nodes [1, 6–8]. These molecules were input into the meta-search engine of protein–protein interaction database String [15], organized, and analyzed as previously described [16]. Interactions identified by experiment, database, neighborhood, gene fusion, co-expression, and co-occurrence were included in the search. A confidence score of 0.15 was used and only direct protein–protein interactions were counted. Due to the limited amounts of mRNA extracted from patient specimens, 70 genes with the highest confidence and interaction scores were maintained in the signaling network for later experiments.

### Quantification of mRNA expression in patients’ specimens and primary chondrocyte cells using quantitative real-time PCR (qRT-PCR)

Conditions of normal and degenerative endplates were confirmed using nuclear magnetic resonance (NMR) analysis, the Modic scoring system, and the Pfriimann disc degeneration grading system. These endplates were removed from a patient’s spine during surgery then rapidly frozen using liquid nitrogen. Total RNAs were extracted using a RNeasy Plus Micro Kit (Qiagen) according to the manufacturer’s instructions, and fold changes were calculated based on mRNA expression levels of the degenerated versus normal endplates, with change thresholds set at 0.8 and 1.2 to pick hits. Primary chondrocyte cells were plated in 6-well dishes, treated, and then harvested. Total RNAs were extracted in TRIzol (Thermo Fisher Scientific) according to the manufacturer’s instructions for qRT-PCR experiments, carried out as previously described [17] using the LightCycler 96 (Roche Diagnostics, Rotkreuz, Switzerland). Primers used are listed in Additional file 1: Table S1. The cycling conditions were: 95 °C for 5 min, 40 cycles of 95 °C for 10 s and 60 °C for 30 s, and then 95 °C for 15 s, 60 °C for 60 s, and 95 °C for 15 s. The fluorescence was measured during 40 cycles of the 60 °C step. Relative mRNA expression was analyzed using the 2^−ΔΔCt^ method.

### shRIPK1-mediated silencing in primary chondrocyte cells

*RIPK1* knockdown was achieved through viral transduction in primary chondrocyte cells using lentiviral transduction particles for shRNAs. The sequences for the short hairpin RNAs for *RIPK1* (shRIPK1) are listed in Additional file 2: Table S2. The shRIPK1s were cloned into the vector pTripz, characterized, and then sequenced. Lentiviral vector packaging and lentiviral transduction were carried out as described previously [18], and shRNA expression was inducted in the presence of doxycycline.

### Overexpression of RIPK1 in primary chondrocyte cells

Full-length cDNA encoding *RIPK1* (NM_001359997.1) was amplified from the *Mus musculus* fibroblast cell line NIH/3T3 (ATCC, USA) using the following primers: 5′-GCTCTAGAGCCACCATGCAACCAGACATGTCCTTGGACA-3′ (*Xba*l) and 5′-TAGGATCCGCTCTGGCTGGCACGAATCAAGTGG-3′ (*Bam*HI). These cDNAs were then cloned into the vector pCDH-EF1-MCS-T2A-Puro (Addgene, Cambridge, MA), characterized, and sequenced. Lentiviral vector packaging and lentiviral transduction were carried out as described previously [18].

### Isolation, culture, and identification of primary chondrocyte cells

Cartilage was obtained from the hind knees of 6- to 10-day-old ICR mice or from IVD degeneration models at the indicated times. After incision of the fiber annulus and removal of the nucleus pulposus with a blade under a dissecting microscope, the translucent endplate cartilage was exposed. The cartilages are shallow, dish-like structures, thin in the center and thick at the boundary. They were minced into 1 mm^3^ pieces with ophthalmic scissors, washed three times with phosphate-buffered saline (PBS) containing 1000 U/mL penicillin and streptomycin 1 mg/L, and then collected aseptically on a sterile bench. Chondrocytes were obtained by digestion with collagenase II, then the cells were washed with PBS and cultured in petri dishes in a humidified incubator containing 5% CO_2_ and 10% O_2_ at 37 °C. Chondrocytes were maintained in Dulbecco’s modified Eagle’s medium (DMEM)/F-12 medium supplemented with 10% fetal bovine serum (FBS), 100 U/mL penicillin and streptomycin 0.1 mg/L). The cells were trypsinized and replated for a few times to purify chondrocytes. Primary chondrocyte cells were identified using Toluidine blue staining. Cells were fixed in 4% formaldehyde and stained with Toluidine blue for microscope observation.

### Cellular senescence assays

Senescence *β*-galactosidase Staining Kits (Cell Signaling Technology) were used for SA-*β*-gal staining according to the manufacturer’s instructions. Cells were cultured in petri dishes at 37 °C in a humidifier incubator containing 5% CO_2_ and 10% O_2_. Briefly, primary chondrocyte cells were fixed with 2% formaldehyde and 0.2% glutaraldehyde, and then incubated with X-gal staining solution. Cells were visualized and imaged with a Nikon Eclipse Ni-U microscope (Nikon, Tokyo, Japan). The percentages of positively stained cells were calculated based on three independent experiments. Chondrocyte senescence was induced using IL-1β at 10 ng/mL for 48 h.

### Animal model of IVD degeneration and histological staining

A total of 18 ICR mouse, aged 6 weeks, were used for caudal vertebra degeneration. Mice were raised in two groups randomly. To develop degeneration, mice received a surgical procedure. Briefly, the mice were anesthetized with 0.3 mL of 0.6% pentobarbital sodium intraperitoneally. The levels between the sixth and seventh, seventh and eighth, and eighth and ninth coccygeal vertebrae were identified under surgical microscope. Induction of degeneration was performed by percutaneous puncture with a 1-mL syringe needle. The needle was introduced until it reached the nucleus pulposus, when it was turned 360° and maintained in the same position for 2 min. The mice were raised in double cages where animals can run through two cages.

The samples were collected at 0, 1, and 3 months after the puncture, and then the animals were executed by neck dislocation. The samples were removed and decalcified in 15% EDTA for a week, then the samples were stored in 10% formaldehyde for 48–72 h, before water flushing for 4 h followed by paraffin embedding. Finally, paraffin sections (3 µm in thickness) were stained with hematoxylin and eosin (H&E) by a standard procedure and imaged at 40–200× magnification (Nikon).

### Apoptosis assays

Primary chondrocyte cells were plated at 6 × 10^5^ cells/dish in 6-well plates and cultured for either 1 week after Lentiviral infection or 48 h of drug treatment. Cells were treated and subjected to apoptosis kit reagents as previously described [17, 19]. Cisplatin (5 μM) was used in this assay as a positive control for methodology. The data were analyzed using FlowJo software (Ashland, OR, USA).

### Western blot assays

The standard procedures used in this study are described previously [16, 19, 20]. Each experiment was independently performed at least three times. Briefly, cartilage cells were lysed in Mammalian Protein Extraction Reagent (MPER™, Thermo Fisher Scientific) and lysis buffer was supplemented with Halt™ Phosphatase Inhibitor Cocktail (Thermo Fisher Scientific) and Complete™ Mini Protease Inhibitor Cocktail (Roche Diagnostics). BCA Protein Assay Kits were used to determine protein concentrations. Proteins were resolved on 4–12% gradient SDS-PAGE gels (Bio-Rad, Hercules, CA) and transferred to PVDF membrane (EMD Millipore, Temecula, CA). Membranes were incubated with primary antibody, followed by HRP-conjugated secondary antibody (Cell Signaling Technology) and signal detected with SuperSignal West Pico Chemiluminescent Substrate (Thermo Fisher Scientific). Immunoblots were quantified using Image J as described. Each experiment was independently performed at least for three times.

### Cytokine assay

Cytokines were measured using the Bio-Plex Pro mouse cytokine 23-plex assay (Bio-Rad) according to the manufacturer’s instructions for the Luminex 200 instrument. Where a value was above or below the reference range, it was assigned the value of the highest or lowest standard, respectively. Lysates were made by homogenizing organs in ice-cold protein DISC lysis buffer (Roche, 30 mM Tris–HCl (pH 7.0), 120 mM NaCl, 10% Glycerol, 1% Triton X-100, complete protease inhibitors) followed by protein level normalization using a BCA assay (Thermo Scientific).

### Statistical analysis

Data are reported as mean ± SD, unless otherwise noted. Significance was analyzed by one-way ANOVA using GraphPad Prism version 5.00 (GraphPad, San Diego, CA, USA), unless otherwise specified [19]. *P < 0.05; **P < 0.01; ***P < 0.001.

## Results

### Signaling network of potential pathology-associated proteins in intervertebral disc diseases

**Fig. 1:**
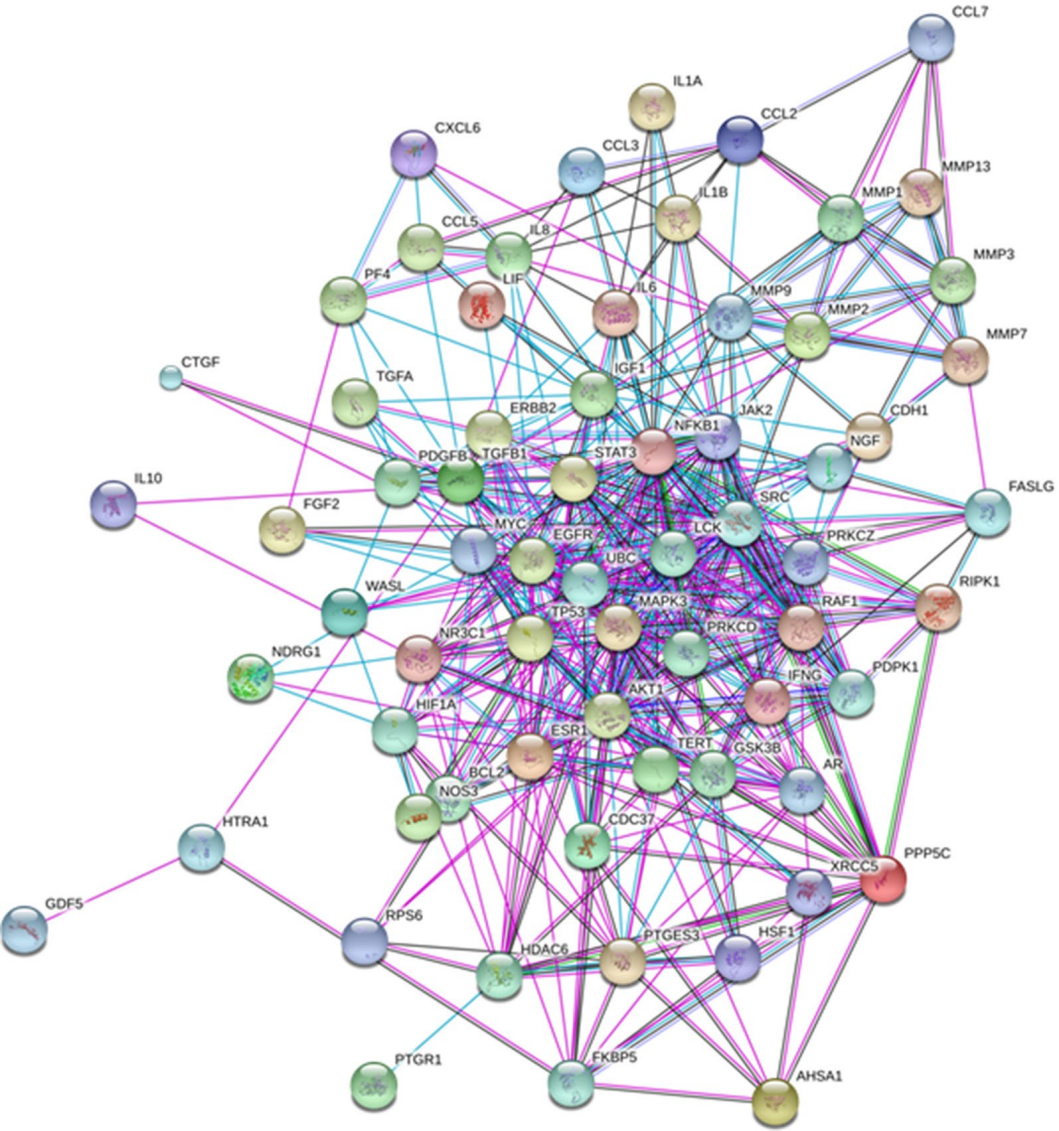
Signaling network of pathology-associated proteins in intervertebral disc degeneration diseases. Reported pathology-associated proteins in intervertebral disc diseases were used as signaling nodes and input into the meta-search engine of protein–protein interaction database String. Interactions from experiment, database, neighborhood, gene fusion, co-expression, and co-occurrence were included in the search. The confidence score of 0.15 was used and only direct protein–protein interactions were counted. We included 70 genes with highest confidence and interaction scores in the signaling network for later experiments. Cyan, pink, green, blue, and black lines indicate known interactions from curated databases, experiments determined, gene neighborhood, gene co-occurrence, and co-expression, respectively. Gene names of these proteins were used in the figure

Building a signaling network is one way to investigate pathology-associated proteins potentially involved in IVD diseases and to identify novel candidates. Changes in load, nutrition, cell metabolism, matrix composition, and matrix turnover can initiate imbalances in IVD homeostasis that alter downstream mediators [1], and some of these might act as signaling nodes in IVD pathologies [1, 6–8]. Potential pathology-associated proteins include growth factors, chemokines, inflammatory proteins, immunomodulatory proteins, cytokines, and proteolytic enzymes, and the gene names of such proteins were submitted for a meta-search of the protein–protein interaction database String. Only direct protein–protein interactions with reasonable confidence levels were included due to the limited amounts of mRNA extracted from patient specimens for qRT-PCR screening. The final built network therefore comprised 70 high-confidence genes with the strongest potential for influencing IVD disease processes (Fig. 1).

### Potential intervertebral disc degeneration-associated proteins

**Fig. 2:**
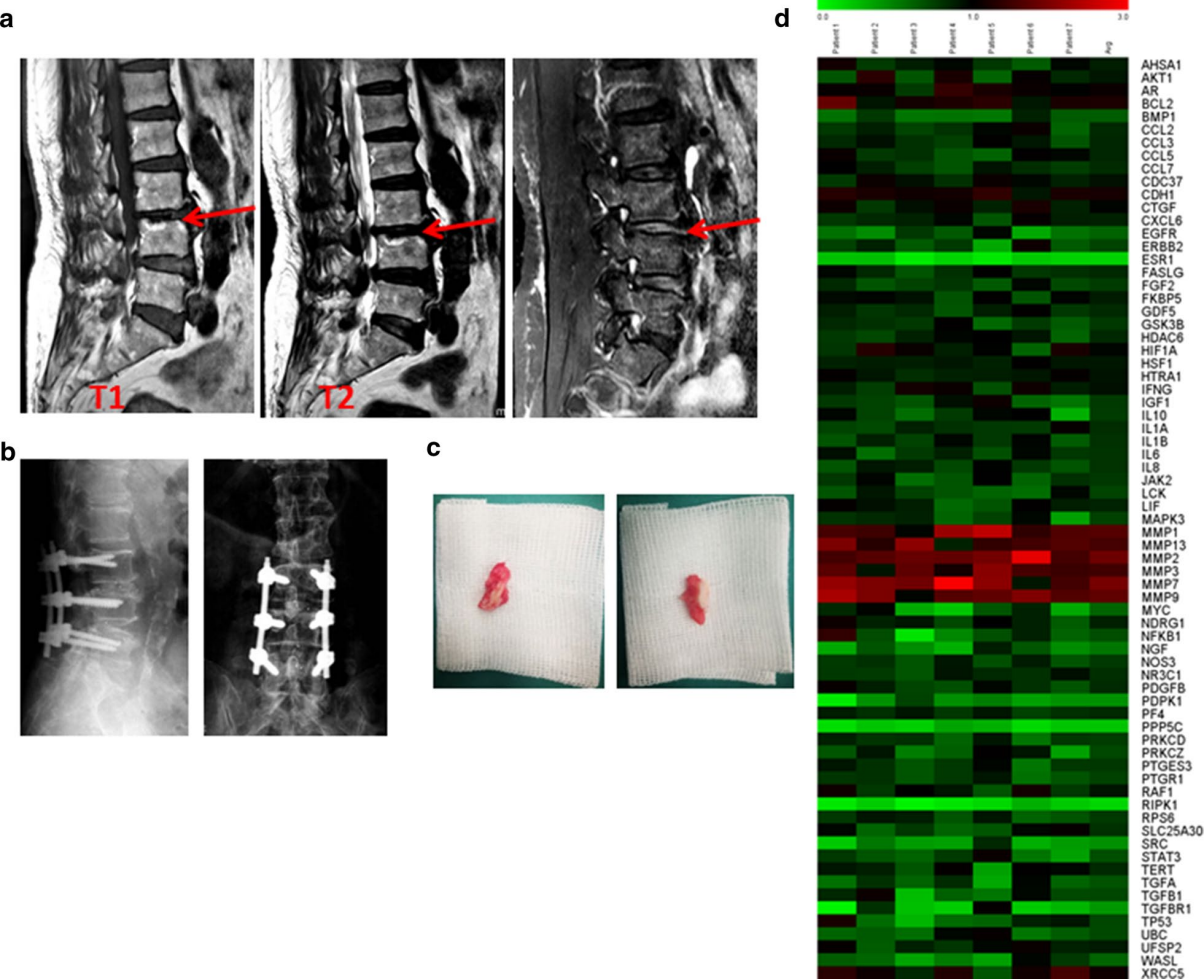
Potential intervertebral disc degeneration-associated proteins identified using patient specimens. **a** Representative NMR images of patients’ spines with Modic grades before the surgery. **b** Representative NMR images of patients’ spines after the surgery. **c** Representative specimen images of normal and degenerative endplates from the patient surgery. **d** Relative mRNA expression of patients’ degenerative versus normal endplates (Patient 1–Patient 7) detected through qRT-PCR experiments with 70 genes of IVD degeneration-associated proteins in the signaling network. Color scale is shown in the corresponding figure

Normal and degenerative endplates were removed from patients’ spines during surgery to form bone transplantation beds to ease fusion and release stress on the spine (Fig. 2a, b). Pairwise degenerated and normal endplates were scored and diagnosed according to the Modic scoring system and the degree of disc degeneration was graded according to the MRI Pfriimann grading system (Table 1, Fig. 2c). Total RNAs were extracted from these endplates and qRT-PCR experiments were performed to evaluate the mRNA expression levels of the signaling network proteins. Fold changes were calculated based on mRNA expression levels of the degenerated versus normal endplates (Fig. 2d). We identified 26 potential IVD degeneration-associated proteins with mRNA changes of more than 20% in degenerated endplates of at least 5 patient specimens (Table 2). As expected, mRNA expression of several (matrix metallopeptidases) MMPs were significantly upregulated, possibly by the production of inflammatory mediators induced via an imbalance in anabolic and catabolic events when degeneration progressed and led to further matrix breakdown and degeneration. Polymorphisms in ESR1, estrogen receptor alpha, have been correlated with bone mass in humans since estrogens are critical for maintaining bone mineral density via diverse mechanisms in osteocytes, osteoclasts, osteoblasts, immune cells, and other cells [21]. The mRNA expression levels of several other genes were significantly changed in the degenerated endplates, including RIPK1, protein phosphatase 5 catalytic subunit (PPP5C), and 3-phosphoinositide dependent protein kinase 1 (PDPK1), none of which have been well studied in bone diseases.

**Table 1:** Patients’ characteristics

No	Age	Sex	Body mass (kg/m^2^)	Diabetes mellitus		Herniation type			Disc degeneration grade (Pfriimann)	Endplate changes (Modic)	Disc height (mm)
				Positive	Negative	Protrusion	Sequestration	Extrusion	(I–IV)	(0–3)	
P1	49	M	22.84		√	√			III	2	14.7
P2	76	F	23.44		√			√	IV	2	14.4
P3	60	M	22.57		√	√	√		IV	2	5.7
P4	72	M	23.56		√	√			III	1	11.1
P5	63	M	27.12		√			√	II	1	15.1
P6	62	M	25.61		√			√	II	1	10.0
P7	46	M	23.12		√			√	II	1	14.9

**Table 2:** Potential intervertebral disc degeneration-associated proteins

Gene name	Official full name	Relevance to IVD disease	References
BCL2	BCL2, apoptosis regulator	NP apoptosis, IVD degeneration	[39]
BMP1	Bone morphogenetic protein 1	OA, osteoblast, osteoclast activities	[40, 41]
EGFR	Epidermal growth factor receptor	IVD degeneration, OA, joint disease/arthritic joints, meniscal injury, post-traumatic OA, articular cartilage, rheumatoid arthritis (RA)	[42–47]
ERBB2	Erb-b2 receptor tyrosine kinase 2	IVD degeneration, arthritis, RA	[48–50]
ESR1	Estrogen receptor 1	Osteoporosis, OA, IVD degeneration	[51–53]
FGF2	Fibroblast growth factor 2	IVD degeneration, disc/bone regeneration, OA	[54–56]
IGF1	Insulin like growth factor 1	IVD, OA, bone formation and growth, bone mineral density	[57–60]
LCK	LCK proto-oncogene, Src family tyrosine kinase	RA	[61]
MMP1	Matrix metallopeptidase 1	Arthritis, IVD, OA, articular cartilage	[62–65]
MMP13	Matrix metallopeptidase 13	Arthritis, OA, IVD degeneration, OA, chondrocytes	[63, 64, 66–68]
MMP2	Matrix metallopeptidase 2	IVD, bone mineralization, joint erosion and defects, OA, RA	[40, 67, 69, 70]
MMP3	Matrix metallopeptidase 3	Cervical spondylosis, IVD degeneration, OA	[63, 71, 72]
MMP7	Matrix metallopeptidase 7	Arthritis, IVD, OA	[73–75]
MMP9	Matrix metallopeptidase 9	Arthritis, IVD, RA, OA	[67, 70, 76, 77]
NFKB1	Nuclear factor NF-kappa-B p105 subunit	OA, IVD degeneration, cervical spondylosis, bone development, osteoporosis	[71, 78–81]
NGF	Nerve growth factor	IVD, OA, bone injury	[82–84]
PDPK1	3-Phosphoinositide dependent protein kinase 1	None	
PPP5C	Protein phosphatase 5 catalytic subunit	None	
PTGR1	Prostaglandin reductase 1	None	
RIPK1	Receptor interacting serine/threonine kinase 1	IVD, arthritis, bone marrow necroptosis	[85–87]
SRC	Proto-oncogene tyrosine-protein kinase Src	Chondrocytes, bone marrow, osteoclast, OA	[88–91]
STAT3	Signal transducer and activator of transcription 3	Bone defect healing, OA, IVD, articular chondrocytes	[92–95]
TGFB1	Transforming growth factor beta 1	OA	[96]
TGFBR1	Transforming growth factor beta receptor 1	OA, articular cartilage	[97, 98]
UBC	Ubiquitin C	IVD, bone destruction and pathologic fracture	[99, 100]
WASL	Wiskott–Aldrich syndrome like	Bone loss or osteoporosis	[101]

### Construction of short hairpin RNAs for *RIPK1* and short-term *RIPK1* knockdown leading to inflammation in primary chondrocyte cells

Abnormal activities of RIPK1 have been indicated in several diseases, including ischemic injuries, chronic and acute inflammatory diseases, and axonal degeneration [14], and it was previously reported that RIPK1 regulates necroptosis and apoptosis. Thus, *RIPK1* was chosen for further investigation with respect to IVD degeneration.

**Fig. 3:**
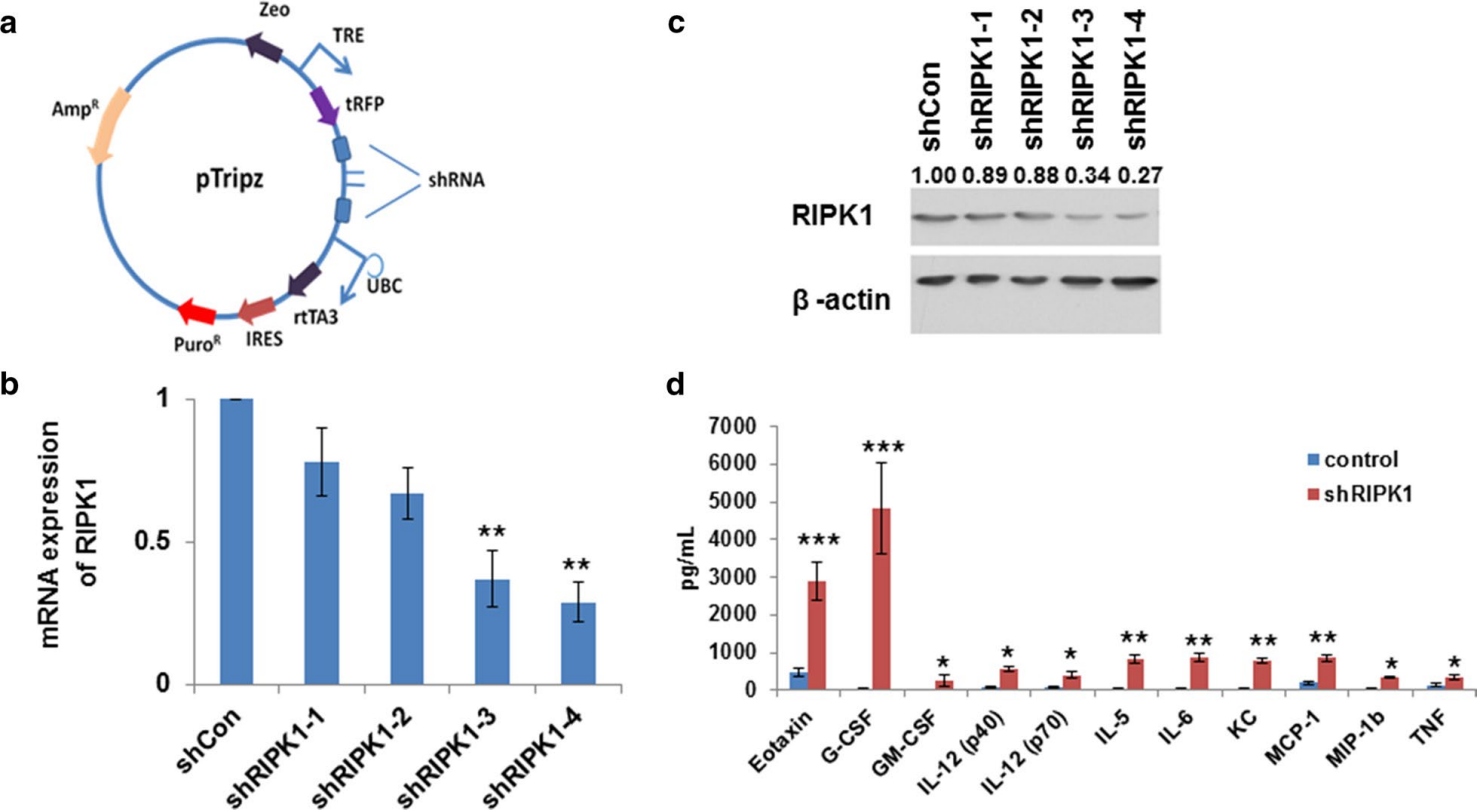
Short-term RIPK1 knockdown led to inflammation in primary chondrocyte cells. **a** Construction of shRIPK1 vectors. **b** mRNA levels of RIPK1 5 days after shRIPK1 knockdown detected by qRT-PCR. **c** Representative Western blot results of shRIPK1 knockdown after 5 days. **d** Inflammatory cytokines levels 5 days after shRIPK1 knockdown. Data indicate the mean values calculated from triplicate samples from multiple independent experiments (n ≥ 3) (± SD). Differences were between shRIPK1 groups and the shControl group. *P < 0.05; **P < 0.01; ***P < 0.001

Vectors of four short-hairpin (sh) RNAs for *RIPK1* were cloned into pTripz as illustrated in Fig. 3a. Primary chondrocyte cells were obtained from 6- to 10-day-old ICR mice and *RIPK*1 knockdown was achieved through viral transduction in primary chondrocyte cells using shRNA lentiviral transduction particles. Knockdown efficiency of these shRNAs for *RIPK1* was tested via qRT-PCR (Fig. 3b) and western blot (Fig. 3c). mRNA expression of *RIPK1* was significantly reduced to 0.37 and 0.29 relative to shRNA controls using shRIPK1-3 and shRIPK1-4, respectively, while protein expression of RIPK1 was significantly reduced to 0.34 and 0.27 relative to shRNA controls using shRIPK1-3 and shRIPK1-4, respectively. These experiments demonstrated that *RIPK1* was successfully and efficiently knocked down with shRIPK1-4, which was therefore chosen for later experiments. RIPK1 has been previously shown to regulate RIPK3-MLKL-driven systemic inflammation [11], thus it was of interest to determine how inflammatory cytokines are regulated in primary chondrocyte cells with *RIPK1* knockdown. Results showed significantly elevated levels of several inflammatory cytokines in primary chondrocyte cells after 4 days of *RIPK1* knockdown by shRIPK1 (Fig. 3d), including Eotaxin, G-CSF, IL5, and MCP-1. These results indicate that inflammation was induced with short-term *RIPK1* knockdown in primary chondrocyte cells.

### Long-term *RIPK1* knockdown leading to apoptosis in primary chondrocyte cells

**Fig. 4:**
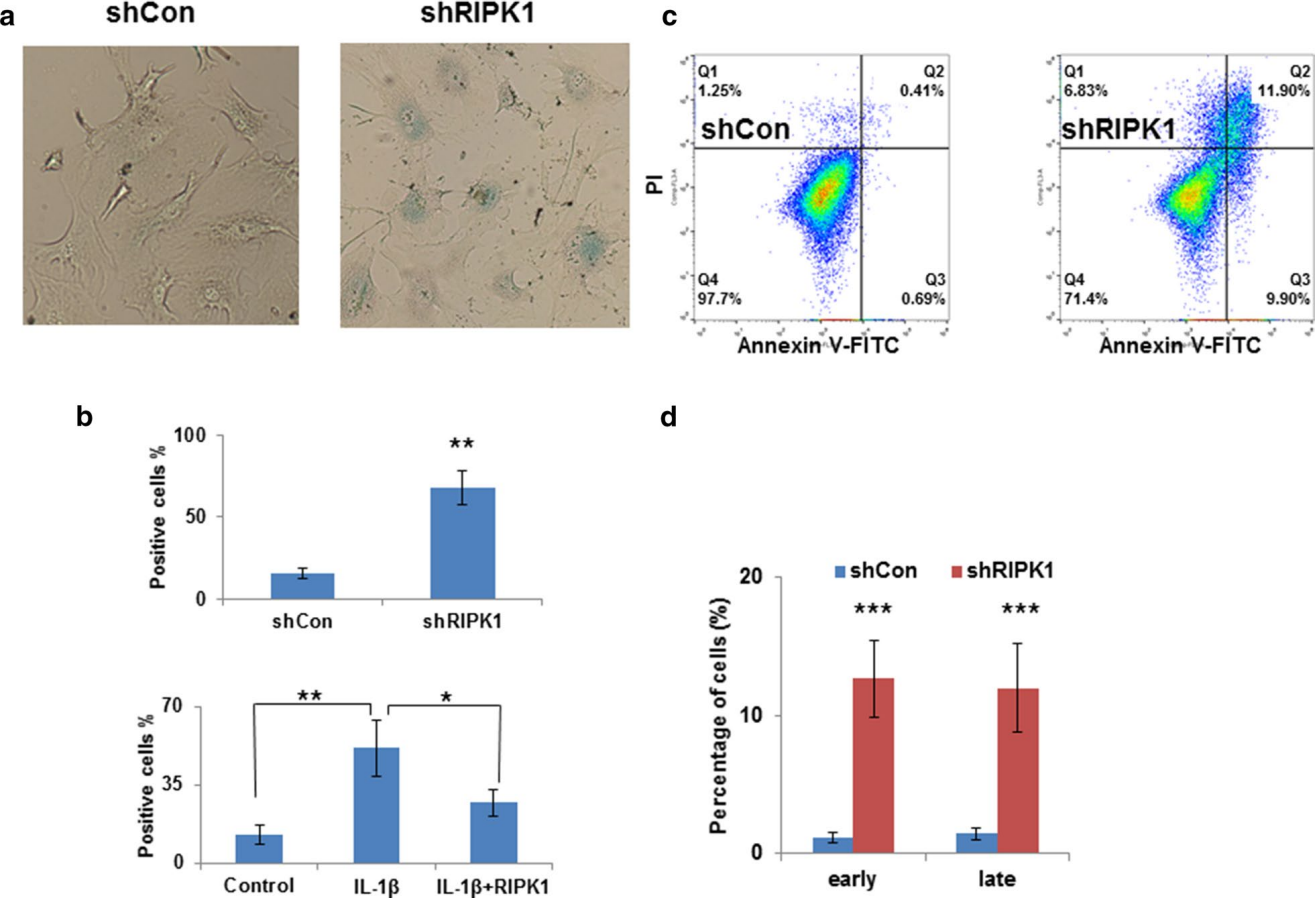
Long-term RIPK1 knockdown led to apoptosis in primary chondrocyte cells. **a** Representative pictures of SA-β-gal staining at 15 days after shRIPK1 knockdown. **b** Statistical analysis of A and of SA-β-gal positive cells in IL-1β induced senescence with or without overexpression of RIPK1. **c** Representative flow cytometry plots for primary chondrocyte cells with RIPK1 knockdown for 15 days. Cells were stained with Annexin V-FITC/propidium iodide and analyzed for cell apoptosis distribution. **d** Statistical analysis of C for the presence of Annexin V (+)/PI (−) (early apoptosis) and Annexin V (+)/PI (+) (late apoptosis)

After 15 days of *RIPK1* knockdown by shRIPK1 in primary chondrocyte cells, there are significantly more senescence phenotypes shown by SA-β-gal assays (Fig. 4a, b; 67.91% increase). IL-1β induced the senescence phenotypes by 51.31%, and this was significantly reversed to 26.97% by RIPK1 overexpression (Fig. 4b). After 15 days of *RIPK1* knockdown, cells were analyzed using flow cytometry (Fig. 4c), indicating that both early and late apoptosis cells were significantly increased after *RIPK1* knockdown (Fig. 4d).

**Fig. 5:**
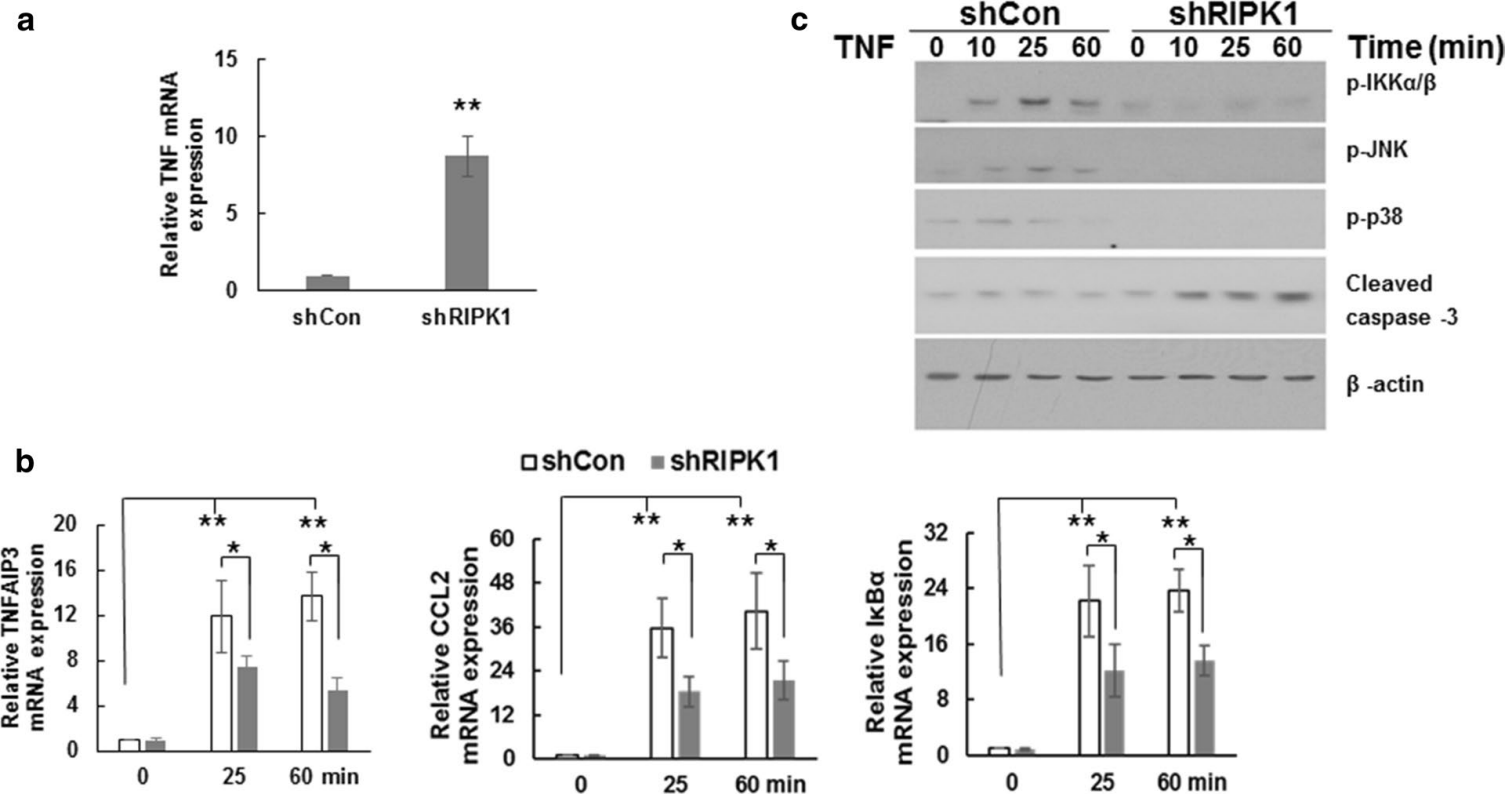
Long-term RIPK1 knockdown triggered apoptosis through the cleaved caspase 3 pathway, while downregulating NF-κB and MAPKs cascades in primary chondrocyte cells. **a** mRNA levels of TNF 15 days after shRIPK1 knockdown detected by qRT-PCR. **b** mRNA levels of TNFAIP3, CCL2, and IκBα 15 days after shRIPK1 knockdown and at the indicated time after TNF stimulation, detected by qRT-PCR. **c** Detection of p-IKKα/β, p-JNK, p-p38, and cleaved caspase 3 in the protein lysates using western blotting. Data indicate the mean values calculated from triplicate samples from multiple independent experiments (n ≥ 3) (± SD). *P < 0.05; **P < 0.01

Additionally, qRT-PCR experiments demonstrated that TNF mRNA expression significantly increased (8.78-fold) after *RIPK1* knockdown (Fig. 5a), prompting us to identify potential apoptosis mechanisms caused by *RIPK1* knockdown and sequential TNF up-regulation. To do this, mRNA levels of NF-κB and MAPK cascades, TNFAIP3, CCL2, and IκBα were assessed following TNF stimulation. Notably, RIPK1 knockdown significantly decreased mRNA levels of TNFAIP3 (11.89 vs. 7.45 at 25 min and 13.67 vs. 5.41 at 60 min), CCL2 (22.16 vs. 12.23 at 25 min and 23.66 vs. 13.64 at 60 min), and IκBα (35.64 vs. 18.34 at 25 min and 40.31 vs. 21.37 at 60 min) after both 25- and 60-min TNF stimulations. Western blotting further proved that protein expression of NF-κB and MAPKs cascades, IKKα/β, JNK, and p38, significantly decreased with *RIPK1* knockdown and TNF stimulation, while protein expression of cleaved caspase-3 increased (Fig. 5c). These results suggest that longer term *RIPK1* knockdown can trigger apoptosis through the cleaved caspase 3 pathway while downregulating the NF-κB and MAPKs cascades and decreasing cell survival and inflammation.

### Mechanisms of intervertebral disc degeneration in animal models

**Fig. 6:**
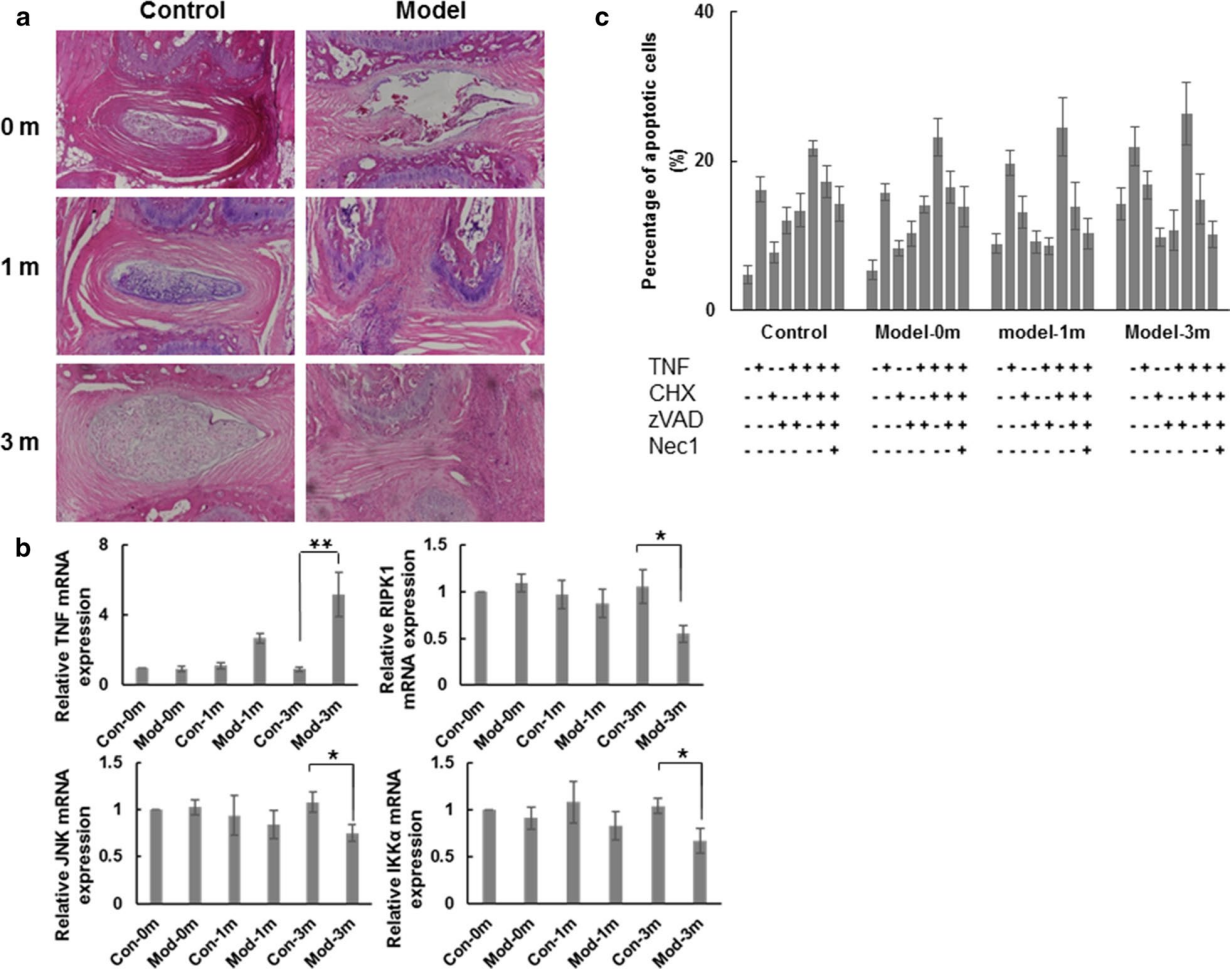
Apoptosis induced in animal models of caudal vertebra intervertebral disc degeneration through TNF-induced caspase-dependent RIPK1 pathway. **a** H&E staining of endplates of animal models of caudal vertebra intervertebral disc degeneration and control group at the indicated time. **b** mRNA levels of TNF, RIPK1, JNK, and IKKα in primary chondrocyte cells from endplates of animal models as indicated. **c** Apoptotic cells of primary chondrocyte cells from endplates of animal models treated as indicated and detected by flow cytometry. Data indicate the mean values calculated from triplicate samples from multiple independent experiments (n ≥ 3) (± SD). *P < 0.05; **P < 0.01

Animal models of caudal vertebra IVD degeneration were used to further assess mechanisms and functions of the *RIPK1* gene. Mice received surgical procedures with needle puncture to induce artificial damage and were raised in double cages to speed up degeneration. Primary chondrocyte cells were collected from the endplates of these animals at the indicated time points and cultured (Fig. 6a). The mRNA expression of TNF, RIPK1, JNK, and IKKα were then assessed in primary chondrocyte cells (Fig. 6b). mRNA levels of TNF were significantly increased by 5.16-fold at 3 months after surgery compared to controls at time 0 and by 5.67-fold compared to controls at 3 months. On the contrary, levels of RIPK1, JNK, and IKKα significantly decreased (by 47.62%, 30.56%, and 35.58%, respectively) at 3 months after surgery compared to controls.

To further investigate the apoptosis occurring in animal models of caudal vertebra IVD and how that might correlate with the mechanism of RIPK1 downregulation, we utilized some tool molecules (Fig. 6c), including cycloheximide (CHX, apoptosis reagent), zVAD (*N*-benzyloxycarbonyl-Val-Ala-Asp (O-Me) fluoromethyl ketone, caspase inhibitor), and necrostatin-1 (Nec1, RIPK1 inhibitor). Results demonstrated that apoptosis was induced by TNF and CHX in RIPK1-downregulated primary chondrocytes of caudal vertebra IVD animal models, but blocked by zVAD. These findings suggested that the apoptosis is dependent on caspase. TNF plus CHX triggered cell death in controls, but more so in caudal vertebra IVD when RIPK1 was downregulated, indicating that RIPK1 suppressed TNF-induced apoptosis. These results are consistent with the earlier findings that RIPK1^−/−^ primary chondrocyte cells are sensitized for cell death induced by TNF plus CHX [22] and that RIPK1 functions to inhibit caspase-mediated apoptosis [9].

## Discussion

IVD degeneration is a complicated issue involving a myriad of factors. Pathological changes have been recognized and classified as ‘degeneration’ as early as in the second decade of life. Recent studies have focused on understanding the molecular and genetic aspects of disc degeneration to diagnosis the degeneration early, identify the optimal time to start therapeutic intervention, and halt or slow down the degenerative process. Nonetheless, apoptosis or programmed cell death appears to correlate with age-related degeneration, with a higher percentage of apoptosis present in older people [23]. Identifying the molecular causes or capturing early events in apoptosis during disc degeneration could revolutionize current treatment of back pain. To this end, the Fas receptor is expressed shortly after the onset of disc degeneration [24], while a high mechanical load, decreased production of important matrix proteins (such as type II collagen and aggrecan), and increased production of degradative, inflammatory, and catabolic molecules (such as TNF, ILs, MMPs, cathepsin, aggrecanase, lysozyme, nitric oxide, and free radicals) are also implicated causally [25].

Since IVD degeneration is a complex disease and there are no prominent therapeutic agents identified to target pathological molecules, the signaling network based on systems biology and protein–protein interaction database analysis might offer new opportunities to implicate targets and biomarkers for IVD degeneration. The signaling network built by us was based on identified pathological molecules as well as proteins not yet implicated in IVD degeneration, but that show interactions with the known pathological molecules in IVD degeneration disease. Since the cells on patient endplates are very limited, we only had enough for qRT-PCR screening to look for differentially expressed mRNAs of the network proteins in degenerated versus normal specimens. Our screening identified ESR1, a known regulator in multiple degenerative and aging diseases such as Alzheimer’s [26, 27], radiographic hip osteoarthritis [28], aging macula disorder [29], and cancers [30]. More importantly, ESR1 has been implicated to play a role in the bone metabolism of osteoporosis, osteocytes, osteoclasts, osteoblasts, immune cells, and other cells to maintain bone mineral density with varieties of mechanisms [21]. Therefore, it was not surprising to detect abnormal expression of ESR1 in patients with IVD disease and it proved that our signaling network and qRT-PCR screening was effective, informative, and identified potential molecules. Another strong hit, PPP5C, inhibits cell growth when it is knocked down in several cell types. We also investigated the strong hit of RIPK1, a mediator of necroptosis, apoptosis, and inflammation [14]. Multiple RIPK1 deficiency studies have shown persuasive evidence that RIPK1 suppresses FADD/caspase-8-dependent apoptosis in some cell types, and RIPK3/MLKL-dependent necroptosis in others [9–12, 31–33]. We showed herein that short-term RIPK1 knockdown can increase inflammatory cytokines, while long-term RIPK1 knockdown led to apoptosis in primary chondrocyte cells. We further demonstrated that long-term RIPK1 knockdown triggered apoptosis through the cleaved caspase 3 pathway while downregulating NF-κB and MAPKs cascades and decreasing inflammation and cell survival. Our capture of both short- and long-term results of RIPK1 knockdown relatively represent the complex and lengthy degeneration process, which in turn seems to be associated with inflammation and infection. Possibly, the impact of apoptosis is more predominant after inflammation and infection to worsen the IVD degeneration, and in animal disease models, the mRNA expression of RIPK1 was lower in degeneration models after 3 months, while that of TNF was significantly higher.

Several caspase proteins have been implicated in the apoptosis of disc degeneration and thus proposed as therapeutic targets [34–36]. The apoptosis we observed is also dependent on caspase as shown by zVAD blockage, and zVAD significantly inhibited apoptosis induced by THF and CHX in primary chondrocyte cells cultured from the animal models. These findings further confirmed that the apoptosis is caspase-mediated and RIPK1 dependent. Our studies suggest that inhibitors of caspase and overexpression of RIPK1 could have therapeutic potential in halting or delaying degeneration in IVD diseases or reversing the IL-1β induced senescence, and thus for developing RIPK1 agitators with low toxicity for the treatment and/or delay of IVD degeneration.

A great number of biomolecular therapies, gene and interfering RNA therapies have been broadly investigated in IVD degeneration. Injection of various growth factors, such as BMPs, EGF, TGF-βs, has shown promising results in delaying degeneration [37]. Viral and non-viral gene delivery of genes, such as Sox-9, OP-1, TIMP-1, and BMP-2, dramatically increased disc height, gene expression, and matrix molecules [37, 38]. Despite harsh environment where these biological molecules are injected and further improvement are required for clinical application, these studies have provided encouraging results about delaying apoptosis and long-term promotion of regeneration. Both natural and synthetic materials can provide favorable scaffolds for tissue engineering and bioactive agent delivery. Natural materials have the advantages such as low toxicity, similarity to native tissue, and easy large-scale production, and synthetic materials are highly reproducible and their mechanical and physicochemical properties can be finely adjusted. All such development will surely contribute to combating IVD degeneration with gene therapy strategies, e.g. gene therapy with *RIPK1*, in the near future.

## Conclusion

These results demonstrated that RIPK1 is a promising IVD degeneration-associated protein and implicated RIPK1-regulated apoptosis in such degeneration. They thus provide a proof-of-concept method for developing novel therapies to combat IVD degeneration through interfering with RIPK1-mediated apoptosis signaling pathways especially in patients with RIPK1 abnormality. Realistically, the goal of our study is not to reverse the disc degeneration completely, but to offer a new insight into the molecular pathology of endplate degeneration and explore the potential to delay the inevitable degeneration.

## Additional files

**Additional file 1: Table S1.** Primer sequences for qRT-PCR experiments.

**Additional file 2: Table S2.** Sequences of shRIPK1s.

## Abbreviations

RIPK1: receptor interacting serine/threonine kinase 1
IVD: intervertebral disc
AF: annulus fibrosus
NP: nucleus pulposus
RIPK: receptor-interacting protein kinase
MLKL: mixed lineage kinase domain like pseudokinase
TLR: Toll-like receptor
TNF: tumor necrosis factor
FADD: Fas-associating protein with a novel death domain
NF-κb: nuclear factor kappa beta
IKKα/β: inhibitor of nuclear factor kappa-B kinase subunit beta
JNK: C-Jun N-terminal kinase
p38: P38 mitogen-activated protein kinases
X-Gal: 5-bromo-4-chloro-3-indolyl-β-d-galactopyranoside
zVAD: *N*-benzyloxycarbonyl-Val-Ala-Asp (O-Me) fluoromethyl ketone
Nec1: necrostatin-1
qRT-PCR: quantitative real-time PCR
ICR: Institute of Cancer Research
Xbal: restriction enzyme isolated from the bacterium *Xanthomonas badrii*
BamHI: (from *Bacillus amyloliquefaciens*) is a type II restriction endonuclease
β-gal: β-galactosidase
IL-1β: interleukin 1 beta
H&E: hematoxylin and eosin
MMP: matrix metalloprotein
ESR1: estrogen receptor alpha
PPP5C: serine/threonine-protein phosphatase 5
PDPK1: phosphoinositide-dependent kinase-1
G-CSF: granulocyte-colony stimulating factor
IL5: interleukin 5
MCP-1: monocyte chemoattractant protein 1
MAPK: mitogen-activated protein kinase
CHX: cycloheximide
