# Supplementary material for: RIPK1 suppresses apoptosis mediated by TNF and caspase-3 in intervertebral discs
Source: J Transl Med. 2019 Apr 27;17:135. doi: 10.1186/s12967-019-1886-3 (PMC6487042; doi:10.1186/s12967-019-1886-3)
Supplement: Supplementary file 1 — Additional file 1: Table S1. Primer sequences for qRT-PCR experiments. [file 12967_2019_1886_MOESM1_ESM.doc]

**Table S1. Primer sequences for qRT-PCR experiments.**

| Gene | Primers | Forward | Reverse | Species |
| --- | --- | --- | --- | --- |
| AHSA1 | Primer1 | ttcacccagggcatgatctt | tggtgacgtcaggaaggttt | H |
|  | Primer 2 | ctggcagtgcaggttcaaaa | atctccacttcatccacgct | H |
| AKT1 | Primer 1 | agcctgggtcaaagaagtca | ccttcacaatagccacgtcg | H |
|  | Primer 2 | attgtgaaggagggttggct | gaaggtgcgttcgatgacag | H |
| AR | Primer 1 | ctctcacatgtggaagctgc | agcctctccttcctcctgta | H |
|  | Primer 2 | cccacttgtgtcaaaagcga | gcagcttccacatgtgagag | H |
| BCL2 | Primer 1 | gccttctttgagttcggtgg | gaaatcaaacagaggccgca | H |
|  | Primer 2 | atccaggataacggaggctg | acttcacttgtggcccagat | H |
| BMP1 | Primer 1 | gacttggccgactacaccta | tgcagctttgatggaggact | H |
|  | Primer 2 | gtcctccatcaaagctgcag | gctaccagtgaagtttcccc | H |
| CCL2 | Primer 1 | tagcagccaccttcattccc | ggtggtccatggaatcctga | H |
|  | Primer 2 | aaagtctctgccgcccttc | cacagatctccttggccaca | H |
| CCL3 | Primer 1 | catcacttgctgctgacacg | gaagcttctggacccctcag | H |
|  | Primer 2 | gctgtcctcctctgcacc | cactcagctccaggtcgc | H |
| CCL5 | Primer 1 | cccatattcctcggacacca | ttgatgtactcccgaaccca | H |
|  | Primer 2 | cgctgtcatcctcattgctac | tgtactcccgaacccatttc | H |
| CCL7 | Primer 1 | aatacttcaactacctgctgct | gtggggtcagcacagatct | H |
|  | Primer 2 | cttcaactacctgctgctaca | gggttttcttgtccaggtgc | H |
| CDC37 | Primer 1 | gggagcagaaacacaagacc | tgcacatttctcctccacct | H |
|  | Primer 2 | ctacagcgtgtgggacca | agtttcctctggcactcgg | H |
| CDH1 | Primer 1 | aacaggatggctgaaggtga | ccttccatgacagacccctt | H |
|  | Primer 2 | ccgattcaaagtgggcacag | ccaggagaggagttgggaaa | H |
| CTGF | Primer 1 | agggcctcttctgtgacttc | gaacgtccatgctgcacag | H |
|  | Primer 2 | ggaagagaacattaagaagggca | gcaggcacaggtcttgatg | H |
| CXCL6 | Primer 1 | cacgaggaaaccaaagtgct | cacagcagagacaggaccag | H |
|  | Primer 2 | ctccacccagctcaggaac | cacctgcagtttaccaatcgt | H |
| EGFR | Primer 1 | aggtgaaaacagctgcaagg | aggtgatgttcatggcctga | H |
|  | Primer 2 | aaaaccggactgaaggagct | tggatcacacttttggcagc | H |
| ERBB2 | Primer 1 | tgtgtgggagctgatgactt | tcttggccgacattcagagt | H |
|  | Primer 2 | catcatctctgcggtggttg | tccgtctctttcaggatccg | H |
| ESR1 | Primer 1 | atgtgcctggctagagatcc | caaactcctctccctgcaga | H |
|  | Primer 2 | caagcccgctcatgatcaaa | tcaaatccacaaagcctggc | H |
| FASLG | Primer 1 | gagaagcaaataggccaccc | ttgcaagattgaccccggaa | H |
|  | Primer 2 | ctgccacccctgaagaagag | atctggctggtagactctcg | H |
| FGF2 | Primer 1 | gccacttcaaggacccca | ggttagcacacactcctttga | H |
|  | Primer 2 | aggagtgtgtgctaaccgtt | cagttcgtttcagtgccaca | H |
| FKBP5 | Primer 1 | tctccttgctgcctttctga | cacccttggctgactcaaac | H |
|  | Primer 2 | agtggggaatggtgaggaaa | ccaatgtcccatgccttgat | H |
| GDF5 | Primer 1 | ccaaacaaggcaggctacag | tgttgcctccctttctgtca | H |
|  | Primer 2 | acaccctccccaaacaagg | catgtactcgtggggtgtga | H |
| GSK3B | Primer 1 | atgtatggtctgctggctgt | cgcactcctgaggtgaaatg | H |
|  | Primer 2 | cccgactaacaccactggaa | ctgtccacggtctccagtat | H |
| HDAC6 | Primer 1 | gcagcttacgggtcatgaag | cttccccaaatgatgccgag | H |
|  | Primer 2 | acctaatcgtgggactgcaa | ttccttttcagcaaaccggg | H |
| HIF1A | Primer 1 | attttggcagcaacgacaca | cagggtcagcactacttcga | H |
|  | Primer 2 | aaagtctcgagatgcagcca | ccaaatcaccagcatccaga | H |
| HSF1 | Primer 1 | cccagcaacagaaagtcgtc | ggagaactgccggctatact | H |
|  | Primer 2 | gcccaagtacttcaagcaca | acacactggtcactttcctct | H |
| HTRA1 | Primer 1 | gcagacatcgcactcatcaa | gcctcccgagtttccatagt | H |
|  | Primer 2 | aacacctacgccaacctgt | tcgatatgaaccacggcagg | H |
| IFNG | Primer 1 | tggaaagaggagagtgacaga | tgtattgctttgcgttggaca | H |
|  | Primer 2 | agcggataatggaactcttttct | agtcagcttttcgaagtcatct | H |
| IGF1 | Primer 1 | atcagcagtcttccaaccca | tgtctccacacacgaactga | H |
|  | Primer 2 | atcgtggatgagtgctgctt | actccctctacttgcgttct | H |
| IL10 | Primer 1 | actgctctgttgcctggtc | acaagttgtccagctgatcc | H |
|  | Primer 2 | agaaccaagacccagacatca | aactcactcatggctttgtaga | H |
| IL1A | Primer 1 | tgaaggctgcatggatcaatc | tgctgacctaggcttgatga | H |
|  | Primer 2 | atcctgaatgacgccctca | gtcttcatcttgggcagtcac | H |
| IL1B | Primer 1 | ctacgaatctccgaccacca | tcagtgatcgtacaggtgca | H |
|  | Primer 2 | tacctgtcctgcgtgttgaa | caggaagacgggcatgtttt | H |
| IL6 | Primer 1 | cagccactcacctcttcaga | accaggcaagtctcctcatt | H |
|  | Primer 2 | gtccagttgccttctccc | gcctctttgctgctttca | H |
| IL8 | Primer 1 | tggcagccttcctgattt | aaccctctgcacccagtt | H |
|  | Primer 2 | ttctgcagctctgtgtgaag | ccagttttccttggggtcca | H |
| JAK2 | Primer 1 | cctcgttggtattgcagtgg | acactgccatcccaagacat | H |
|  | Primer 2 | tacaggacaacactggggag | ttagattacgccgaccagca | H |
| LCK | Primer 1 | ccccttcaattttgtggcca | agccaccgttgtccagatta | H |
|  | Primer 2 | cggagatctgggctttgaga | gatgaggaaggagccgtgag | H |
| LIF | Primer 1 | tgaaccagatcaggagccaa | gccaaggtacacgactatgc | H |
|  | Primer 2 | ccagaagatcctcaacccca | acacggcgatgatctgctta | H |
| MAPK3 | Primer 1 | atcttccctggcaagcacta | agggctttggagtctgactt | H |
|  | Primer 2 | atttgtgatttcggcctggc | atccaggtagtgcttgccag | H |
| MMP1 | Primer 1 | aaggtctctgagggtcaagc | tcatgagctgcaacacgatg | H |
|  | Primer 2 | atagtggcccagtggttgaa | ggctttctcaatggcatggt | H |
| MMP13 | Primer 1 | aaggagcatggcgacttcta | aaacatgagtgctccagggt | H |
|  | Primer 2 | gactgagaggctccgagaaa | tcggaccaaactttgaaggc | H |
| MMP2 | Primer 1 | ggtccgtgtgaagtatggga | aagcggaatggaaacttgca | H |
|  | Primer 2 | caagtctggagcgatgtgac | atcggcgttcccatacttca | H |
| MMP3 | Primer 1 | aatcctactgttgctgtgcg | catcacctccagagtgtcgg | H |
|  | Primer 2 | attggaggtgacggggaag | acagcctggagaatgtgagt | H |
| MMP7 | Primer 1 | aaactcccgcgtcatagaaa | tgtcagcagttccccataca | H |
|  | Primer 2 | acaggctcaggactatctca | tgagtattctgcaacatctggc | H |
| MMP9 | Primer 1 | cctggagacctgagaaccaa | tggaatctgcccaggtctg | H |
|  | Primer 2 | accctgccagtttccattca | ggtcgagtactccttacccag | H |
| MYC | Primer 1 | attctctgctctcctcgacg | ctgtgaggaggtttgctgtg | H |
|  | Primer 2 | ggacgacgagaccttcatca | cgttgagagggtaggggaag | H |
| NDRG1 | Primer 1 | aggcgcctacatcctaactc | ccggggttcatgtcattcac | H |
|  | Primer 2 | actacgaggacatgcaggag | tcgagttaggatgtaggcgc | H |
| NFKB1 | Primer 1 | tgtccagcttcggaggaaat | cactaccaaacatgcctccg | H |
|  | Primer 2 | gtggagcacgacaacatctc | tccaggcacaactccttcat | H |
| NGF | Primer 1 | ggtgaagcctttggtggaga | atgccgatgtcatggtaggt | H |
|  | Primer 2 | tgcttcctggagtccttcaa | ggtccatcctgagatcttgga | H |
| NOS3 | Primer 1 | agagtggacgcacagtaaca | ccacctcccagttcttcaca | H |
|  | Primer 2 | accctcaccgctacaacatc | gctcattctccaggtgcttc | H |
| NR3C1 | Primer 1 | tttccctcctgctccttctg | tcacatctcccctctcctga | H |
|  | Primer 2 | tcaggagaggggagatgtga | tcccaggtcatttcccatca | H |
| PDGFB | Primer 1 | caccaacgccaacttcctg | actgtctcacacttgcatgc | H |
|  | Primer 2 | aagggcaagcaccggaaatt | atcactccaaggaccccatg | H |
| PDPK1 | Primer 1 | ttgttgttggagaagcaggc | attcttggcctctggtcgaa | H |
|  | Primer 2 | caggacgacgagaagctgta | ccggtttaaggtccctgtga | H |
| PF4 | Primer 1 | ccagcccgagtttcccat | cggccttgatcacctcca | H |
|  | Primer 2 | tgaagctgaagaagatgggga | cacacacgtaggcagctagt | H |
| PPP5C | Primer 1 | tgaagccccatgacaaggat | cactttgccgtcttcaagct | H |
|  | Primer 2 | acacccatggccagttctat | ccttcacctcaccctcgaaa | H |
| PRKCD | Primer 1 | tccaccccttcttcaagacc | cgatgaggttcttgtcgctg | H |
|  | Primer 2 | ctgtgccgtgaagatgaagg | attgttcttcttgcagcgct | H |
| PRKCZ | Primer 1 | cgacatcatcaccgacaacc | tcttctccagcaagtcccag | H |
|  | Primer 2 | ctgggacttgctggagaaga | cggtggacagcaataatggg | H |
| PTGES3 | Primer 1 | ctgagggaagcgagaagagg | gtcccttcgatcgtaccact | H |
|  | Primer 2 | acattcagttgtctcggagga | gccaattaagctttgccctt | H |
| PTGR1 | Primer 1 | agttgttggagcagtagggt | aagtgggccggttctgttat | H |
|  | Primer 2 | gctgagctcccacccttaaa | tgtcagcagcttttccagatc | H |
| RAF1 | Primer 1 | tgtccagtagccccaacaat | tctccgtgccatttaccctt | H |
|  | Primer 2 | ccagctgcatctctcctaca | aagtctgaacactgcacagc | H |
| RIPK1 | Primer 1 | agtgacttcctggagagtgc | tcatcatcttcgcctcctcc | H |
|  | Primer 2 | aagccgagatgagtactccg | gtccacttccctcagctcat | H |
| RPS6 | Primer 1 | tgacgctctgggtgaagaat | catccacaatgcaaccacga | H |
|  | Primer 2 | agagctagcagaatccgcaa | cttggtacgctgcttcttca | H |
| SLC25A30 | Primer 1 | agacacggctccagattcaa | tgcctatcttgatggtgcca | H |
|  | Primer 2 | taccagcaagaggggacaag | aagcttgagaggaagtgggt | H |
| SRC | Primer 1 | aaaccagatgaggacgctga | gctcttgttgctacccatgg | H |
|  | Primer 2 | cacaggacagacaggctaca | tccagcttgcggatcttgta | H |
| STAT3 | Primer 1 | agaaggaggcgtcactttca | tttccgaatgcctcctcctt | H |
|  | Primer 2 | agaaaacatggctggcaagg | gcctccttctttgctgcttt | H |
| TERT | Primer 1 | agccacgtctctaccttgac | ggcacatgaagcgtaggaag | H |
|  | Primer 2 | cttcctacgcttcatgtgcc | aatcatccaccaaacgcagg | H |
| TGFA | Primer 1 | cccagattcccacactcagt | acctggcagcagtgtatcag | H |
|  | Primer 2 | cgctctgggtattgtgttgg | gcaccaacgtacccagaatg | H |
| TGFB1 | Primer 1 | atcgacatggagctggtgaa | ctccttggcgtagtagtcgg | H |
|  | Primer 2 | cagcagggataacacactgc | tgcggaagtcaatgtacagc | H |
| TGFBR1 | Primer 1 | cctcgagataggccgtttgt | acagcaacttcttctccccg | H |
|  | Primer 2 | ccgtttgtatgtgcaccctc | gtgaatgacagtgcggttgt | H |
| TP53 | Primer 1 | gcccctcctcagcatcttat | aaagctgttccgtcccagta | H |
|  | Primer 2 | aggttggctctgactgtacc | gattctcttcctctgtgcgc | H |
| UBC | Primer 1 | tagtcccttctcggcgattc | acgaagatctgcattgtcaagt | H |
|  | Primer 2 | agaaccccagtatcagcaga | gcgtccttatataatcatcggcg | H |
| UFSP2 | Primer 1 | gctggaaatgtcaacctccc | aaaagtgcagtggttcaggg | H |
|  | Primer 2 | actcctggttgatgcaattca | aatagggtctgtcgtgagg | H |
| WASL | Primer 1 | tctggacgagatgcactgtt | tggctttgctccttttctgc | H |
|  | Primer 2 | tgtggaatctcagaggcaca | gaggacctgagttgtgtgga | H |
| XRCC5 | Primer 1 | gcaagagatgatgaggcagc | gccgcaagtcttccatgaaa | H |
|  | Primer 2 | cgtgagcatggatgtgattca | catctcctctgtccccactt | H |
| RIPK1 | Primer 1 | aaggataatcgtggaggcca | tgctgctcacttctttctgc | M |
|  | Primer 2 | actgtgtacccttacctccg | gcatgatagctggcttcctc | M |
| TNF | Primer 1 | agcacagaaagcatgatccg | ctgatgagagggaggccatt | M |
|  | Primer 2 | aatggcctccctctcatcag | agccttgtcccttgaagaga | M |
| TNFAIP3 | Primer 1 | actcggaactggaatgacga | ccacccactttcaaaggagc | M |
|  | Primer 2 | acgagagagaaccccagaag | agcttcctgacttcacgaca | M |
| CCL2 | Primer 1 | accagcaagatgatcccaatg | cgggtcaacttcacattcaaag | M |
|  | Primer 2 | gtctgtgctgaccccaag | atcacagtccgagtcacact | M |
| IκBα | Primer 1 | agcagactccactccacttg | acgtgtggccattgtagttg | M |
|  | Primer 2 | acgagcaaatggtgaaggag | tcccttcacctgaccaatga | M |
| JNK | Primer 1 | tgcaatcaagaagctcagcc | gatttgcatccatgagctcca | M |
|  | Primer 2 | tcggaacaccttgtcctgaa | cgttgatgtatgggtgctgg | M |
| IKKα | Primer 1 | aacgtcagtctgtaccagca | cccctccagaacagtactcc | M |
|  | Primer 2 | ataagccgtacacagccact | tgtttggctgaggtaaatggc | M |

Species: H-homo sapiens

M-mus musculus
