# Supplementary material for: RIPK1 suppresses apoptosis mediated by TNF and caspase-3 in intervertebral discs
Source: J Transl Med. 2019 Apr 27;17:135. doi: 10.1186/s12967-019-1886-3 (PMC6487042; doi:10.1186/s12967-019-1886-3)
Supplement: Supplementary file 2 — Additional file 2: Table S2. Sequences of shRIPK1s. [file 12967_2019_1886_MOESM2_ESM.doc]

**Table S2. Sequences of shRIPK1s.**

|  | Forward | Reverse |
| --- | --- | --- |
| shRIPK1-1 | 5’-TCGACCTGAATGACATCAATGCAAACTCGAGTTTGCATTGATGTCATTCAGGTTTTT-3’ | 5’-AATTAAAAACCTGAATGACATCAATGCAAACTCGAGTTTGCATTGATGTCATTCAGG-3’ |
| shRIPK1-2 | 5’-TCGACGTGACTTTCACATTAAGATACTCGAGTATCTTAATGTGAAAGTCACGTTTTT-3’ | 5’-AATTAAAAACGTGACTTTCACATTAAGATACTCGAGTATCTTAATGTGAAAGTCACG-3’ |
| shRIPK1-3 | 5’-TCGAGCATTGTCCTTTGGGCAATATCTCGAGATATTGCCCAAAGGACAATGCTTTTT-3’ | 5’-AATTAAAAAGCATTGTCCTTTGGGCAATATCTCGAGATATTGCCCAAAGGACAATGC-3’ |
| shRIPK1-4 | 5’-TCGAGCCAAATCTAAGCCAAATGTACTCGAGTACATTTGGCTTAGATTTGGCTTTTT-3’ | 5’-AATTAAAAAGCCAAATCTAAGCCAAATGTACTCGAGTACATTTGGCTTAGATTTGGC-3’ |
